# The Peculiar People and the Law

**Published:** 1902-04-19

**Authors:** 


### The Peculiar People and the Law.

Two members of that curious sect the Peculiar People were charged last week at the Central Criminal Court with the manslaughter of their son, a boy who, being taken ill with a cold which turned out to be pneumonia, in the end died without medical attendance. In accordance with the custom of their sect the parents did not send for a doctor, but nursed the child and called in an elder who anointed him with oil. The defendants, it was stated, were most respectable people, and did everything possible for the child except calling in a doctor, and they maintained in their defence that they had personally experienced the efficacy of prayer and of the anointing with oil. They were, however, found guilty and were sentenced, the man to four months', and the woman to two months' imprisonment in the second division.

To all who have at heart the well-being of children it will be satisfactory to find that medical attendance is still regarded as a "necessary" which must be provided by those who are responsible for their care. At the same time one cannot but feel that the law is easily satisfied, and that so far as protection of child life is concerned the conviction of a few Peculiar People not only goes but a very little way into a very large question, but is in curious contrast with the laxity which prevails in other directions. It is sufficiently obvious that if these people had but taken this unfortunate child to a doctor, though so taking him might have been greatly to his detriment, and even though they had thrown the doctor's medicine down the sink and his recommendations to the dogs, no one would have had a word to say against them, and it is difficult to avoid drawing a comparison between the punishment meted out to them, and the general approbation which is given, both by law and custom, to many who deal with their children far less tenderly. There are few sadder sights in London than that of women carrying helpless infants through the slush and snow, or perchance standing outside hospital gates, exposing their wretched charges to cold and wind and thus taking away from them their last little chance of life, and all this on the plea of "seeing the doctor," the real object of this visit being not the relief of the child, but the gaining of that talisman, the "certificate," which, when the baby dies, is to save them "trouble" with the coroner. It is hard to have patience with a system which, at a time when warmth and food and care are most essential, practically compels a perfunctory visit to the doctor, which imprisons parents who refuse to conform to this custom, and yet at the same time exhibits such indifference to infant life as is displayed in all our arrangements regarding the registration and burial of those who die. If the prosecution of these Peculiar People were part of a general scheme to render compulsory the proper medical certification of all deaths it would have some meaning. But this is not the case, for side by side with it we find a system in full swing which enables callous baby-farmers and hard-hearted, or may be broken-hearted, mothers, by aid of a certificate, often obtained literally at the cost of the infant's life, to comply with all the demands of society, and to fulfil all the requirements of the law. No one can feel more strongly than we do that good medical attendance ought to be provided for even the poorest child when ill. At the same time, living as we do in the midst of the sights which meet the eye at every turn in London, one cannot help asking whether this repeated prosecution of the Peculiar People is not an example of misdirected zeal. Knowing as one does the terms on which the gin-sodden parents of a well-insured infant can get rid of their charge while conforming with every requirement of the law, and the very small protection which the law affords to children whose lives are really endangered by the cruelty and neglect of those in charge of them, one doubts the expediency of these prosecutions of people who admittedly do everything they can except that one thing—the provision of medical attendance. Hard cases, we are told, make bad law; and the repeated spectacle of decent and otherwise law-abiding people going to prison rather than call in a doctor—a proceeding which may be so twisted as to become a mere shibboleth—is of all things the most likely to lead to an alteration of what, after all, is a good and useful provision of the law, namely, that among the necessaries which those in charge of helpless people must provide for them is medical attendance.

